# Hereditary Spherocytosis With Liver Transplantation After Cirrhosis: A Case Report

**DOI:** 10.3389/fmed.2022.823724

**Published:** 2022-02-11

**Authors:** Xueliang Yang, Wen Wang, Wanhu Fan, Lin Cai, Feng Ye, Shumei Lin, Xiaojing Liu

**Affiliations:** ^1^Department of Nutrition, The First Affiliated Hospital of Xi'an Jiaotong University, Xi'an, China; ^2^Department of Infectious Diseases, The First Affiliated Hospital of Xi'an Jiaotong University, Xi'an, China; ^3^Department of Pathology, Shaanxi Provincial Cancer Hospital, Xi'an, China

**Keywords:** hereditary spherocytosis, next-generation sequencing, cirrhosis, liver transplantation, SPTB

## Abstract

The clinical manifestations of hereditary spherocytosis are similar to those of various hemolytic anemias, which causes hereditary spherocytosis to be difficult to diagnose clinically. In this case, we obtained the peripheral blood of a patient and family members, and through a whole exome test of the 6,297 genetic phenotypes confirmed by OMIM, we found a heterozygous nonsense mutation (c.4117C>T, P.Q1373X) in the SPTB gene. Combined with the patient's clinical data, the diagnosis was hereditary spherocytosis. Compared with the public population sequence database, the mutation was found to be unique. Through protein structure prediction analysis and literature studies, we found that the mutation may cause SPTB mRNA instability, resulting in insufficient spectrin protein synthesis and affecting the integrity and flexibility of the red blood cell membrane skeleton. This case report found that SPTB gene mutations may cause liver dysfunction and cirrhosis in addition to hereditary spherocytosis, and this finding expands the phenotypic spectrum of SPTB. This study confirmed that NGS can be used to diagnose hereditary spherocytosis. Identifying mutated genes can not only accurately treat diseases, but also avoid potential genetic risks and improve prenatal and postnatal care.

## Introduction

Hereditary spherocytosis (HS) is a hereditary non-immune hemolytic disease that is characterized by the presence of spherical erythrocytes in peripheral blood smears (1). The prevalence of neonatal HS in northern European and North American countries is ~1/5000 and 1/2000, respectively, and the prevalence of neonatal HS in China is ~0.18/1 million (2). The clinical presentation of HS varies widely from asymptomatic patients to severe anemia, jaundice, and hepatosplenomegaly. The diagnosis of HS is based primarily on clinical manifestations such as jaundice, splenomegaly, and gallstones, with laboratory examination confirming the presence of extravascular hemolysis (3). The above presentation is also common in thalassemia, thus, the two diseases can be easily confused.

HS is most commonly associated with autosomal dominant inheritance, and about 75% of HS cases are autosomal dominant. However, about 25% of patients have no family history, which may reflect autosomal recessive inheritance or newly emerged mutations (4). Patients with recessive inheritance usually have severe symptoms, such as severe life-threatening hemolysis (5, 6). The molecular pathogenesis of HS is due to gene mutations that cause defects in the anchor protein, spectrum protein, band 3, or protein 4.2, which constitute the erythrocyte membrane and lead to reduced denaturation of red blood cells and extrascular-hemolysis (7–9). Mutations in the *ANK1* (Anchor 1), *SPTB* (Spectrin, Beta, erythrocytic), *SPTA1* (Spectrin, alpha, erythrocytic 1), *SLC4A1* (Solutic-carrier family 4, Member 1), and *EPB42* (erythrocyte membrane protein band 4.2) genes have been found to cause HS (10–13). HS is mainly due to mutations in *ANK1* and *SPTB* genes in Chinese individuals (14).

Next-generation sequencing (NGS) is a high-throughput and cost-effective sequencing technology that is capable of performing whole-exome sequencing. NGS mainly focused on the clinical diagnosis of genetically related diseases and facilities the discovery of potential pathogenic or mutant genes (15). This study reports a case of HS with liver transplantation after cirrhosis, summarizes its clinical manifestations, laboratory test results, and gene sequencing data, and conducts a bioinformatics analysis to improve our understanding and diagnoses of HS and our knowledge of the clinical outcomes of SPTB gene mutations.

## Case Presentation

**Figure 1:**
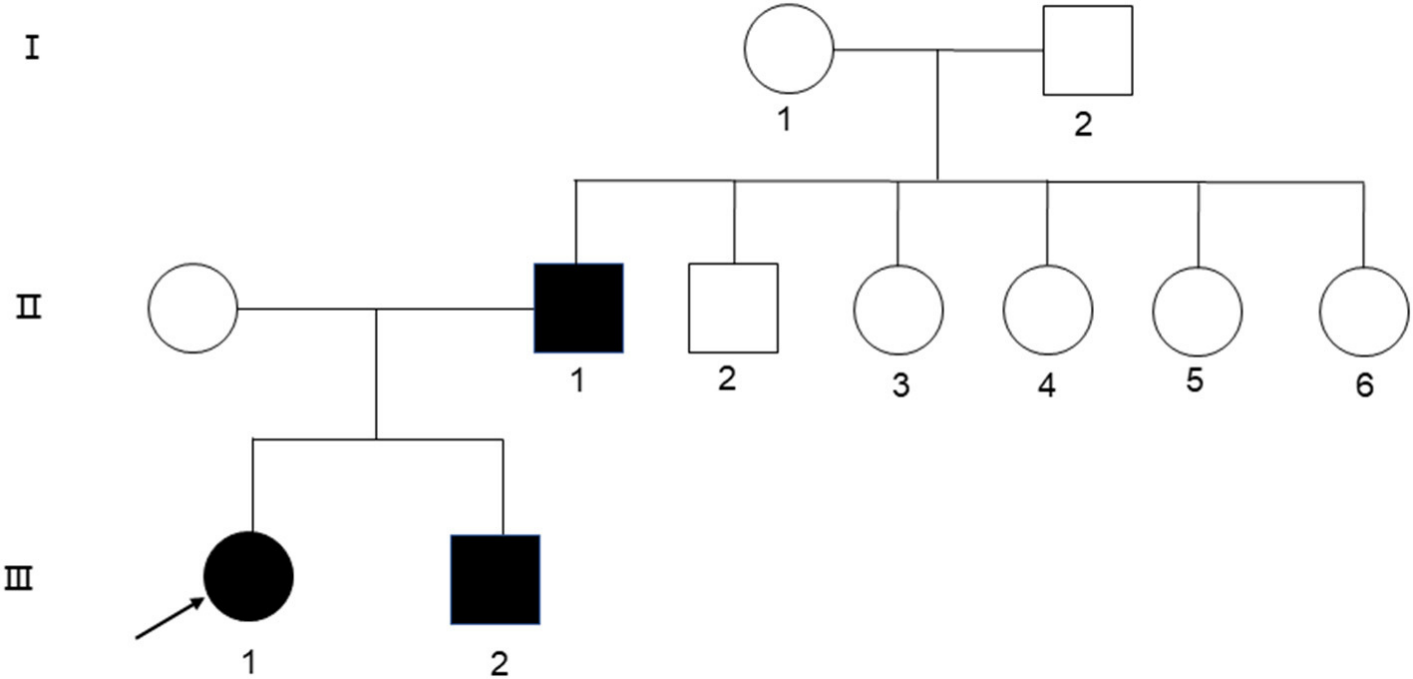
Family diagram of three patients (II1, III1, III2; the arrow shows the proband).

A 24-year-old woman presented at the First Affiliated Hospital of Xi'an Jiaotong University in September with jaundice for 24 years and liver cirrhosis for one year. She had been diagnosed with non-autoimmune hemolytic anemia 24 years ago, based on a medical history of persistent anemia and hyperbilirubinemia. Eight years later, she underwent splenectomy due to refractory anemia, severe jaundice, and splenomegaly. At 22 years of age, the jaundice reappeared with abdominal distension, liver discomfort, fatigue, anorexia, and fever. Laboratory tests showed a hemoglobin level of 110 g/L, platelet count of 198 × 10^9^/L, reticulocyte ratio of 15%, aspartic transaminase level of 87.8 U/L, glutamic transaminase level of 71 U/L, total bilirubin level of 127 μmol/L, and albumin level of 29.6 g/L. Ferritin level of 325 ng/ml. Peripheral blood smear tests showed visible round and densely colored spherical red blood cells, which lacked pale white centers and presented smaller diameters than normal red blood cells. A hemoglobin peptide chain analysis revealed no abnormalities. Hemolysis reaction, red blood cell osmotic fragility test, and G6PD activity were normal. Both the father and younger brother suffered from hemolytic anemia, and 16 years ago, her younger brother underwent splenectomy due to hemolytic anemia (Figure 1).

**Figure 2:**
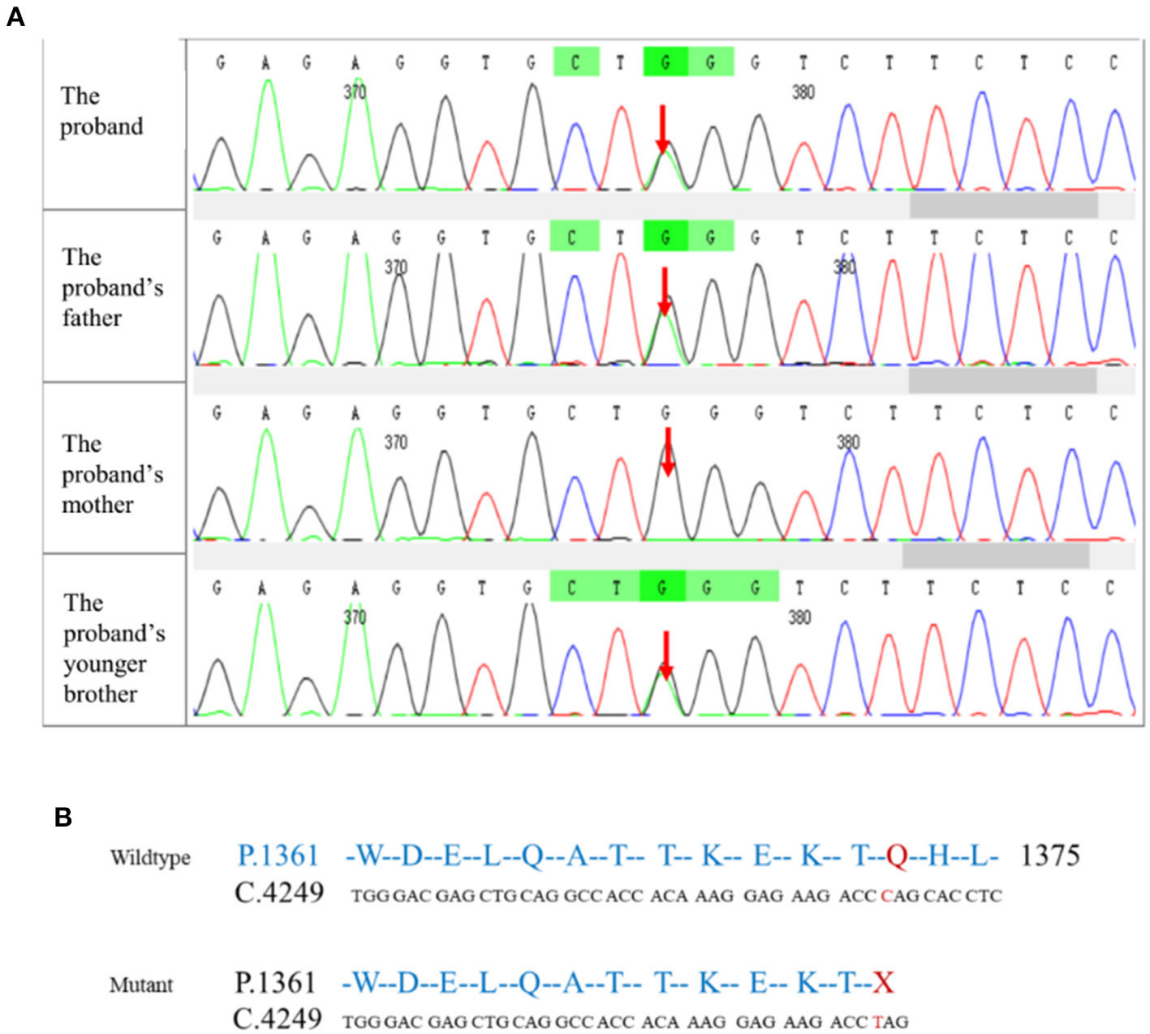
Mutation testing analysis of the SPTB amino acid sequence. **(A)** NGS correlation peak map; **(B)** Wildtype and mutant SPTB sequences.

To identify genetic factors of the disease, the peripheral blood of the proband, the proband's parents, and the proband's younger brother were obtained. The 6,297 genetic phenotypes currently confirmed by OMIM were tested through a whole exome NGS analysis that focused on a total of 412 genes related to the subject's clinical phenotype (Wuhan Kindstar Diagnostics Co., Ltd, Wuhan, China). As a result, one heterozygous nonsense mutation, c.4117C>T, p.Q1373X (reference sequence: NM_001355436), was identified in exon 20 of the proband's *SPTB* gene, the proband's parents, and the proband's younger brother (Figure 2).

**Figure 3:**
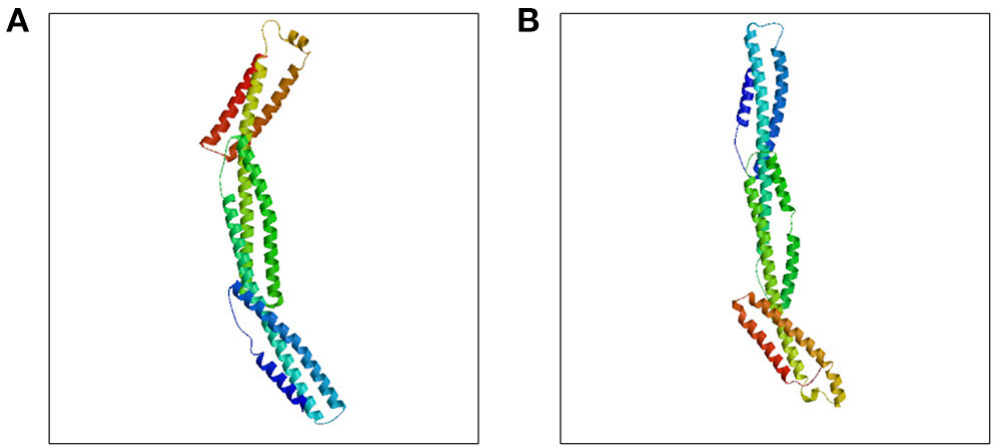
Prediction of SPTB protein structure by Swiss-model. **(A)** Wild type SPTB, **(B)** Mutant SPTB.

*SPTB* gene abnormalities lead to hereditary spherocytosis type 2 with autosomal dominant inheritance (OMIM#616649). In this test, we detected a nonsense variant in the *SPTB* gene of the proband that was inherited from her father, and the father and younger brother of the proband carried the same heterozygous variant. In autosomal dominant diseases, a single pathogenic variant can cause disease. The mutation was not found in public population sequence databases, including HGMD, 1,000 Genomes Project Database, ESP6500, ExAC, and dbSNP. The mutation sequence was predicted in SWISS-MODEL, and it was found that the mutation of the β-spectrum protein p.Q1373X caused the early termination of the protein and subsequently triggered the loss of α-helix (Figure 3). Based on clinical symptoms, laboratory tests, and NGS sequencing, the proband was diagnosed with HS.

The hepatitis virus antibody, autoantibody, and autoimmune liver disease antibody screening were all negative. Furthermore, Epstein-Barr virus DNA and cytomegalovirus DNA were both negative. Screening for metabolic disorders such as hemochromatosis and hepatolenticular degeneration were normal, and no long-term heavy drinking, nutritional disorders, or exposure to industrial poisons or drugs were noted. With regards to 16,569 sites in the patient's mitochondrial gene, no abnormalities were found in 66 mutations that have been reported to cause disease. Re-analysis of the data of all exons did not reveal a second single-gene disease.

Later, she experienced severe upper gastrointestinal bleeding accompanied by hepatic encephalopathy and massive ascites, and her hemoglobin level was at least 50 g/L. Laboratory tests showed an aspartic transaminase level of 65 U/L, glutamic transaminase level of 44 U/L, total bilirubin level of 213.3 μmol/L, albumin level of 33 g/L, plasma ammonia level of 97 μmol/L, creatinine level of 64 μmol/L, INR of 1.67, and So MELD score of 22. Gastroscopy showed esophageal varices (Lemi D 2 Rf1), and computed tomography of the abdomen revealed liver cirrhosis, portal hypertension, massive abdominal and pelvic effusion, thickening of the gallbladder wall, and bilateral pleural effusion. Liver computed tomography angiography images showed a thin hepatic artery, portal hypertension, opening of the left gastric vein, and varicose veins in the lower part of the esophagus and stomach, which presented multiple nodules in the liver, with low-density shadows in the right leaf of the liver. Liver magnetic resonance imaging showed multiple patchy abnormal enhancements in the liver during the dynamic phase, and some of the cirrhotic nodules could be malignant. A liver biopsy was not performed because of coagulopathy.

**Figure 4:**
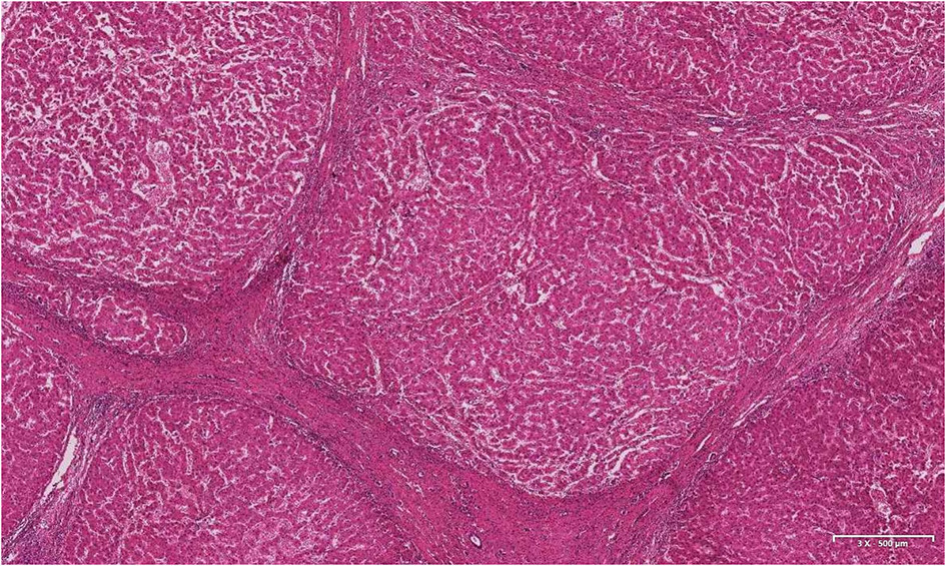
Hepatectomy specimen pathology.

The patient underwent classic orthotopic liver transplantation and portal vein thrombectomy in October 2019. Histopathological examination of the resected specimens revealed nodular liver cirrhosis and chronic cholecystitis. The portal vein thrombosis pathology showed thrombosis with organization (Figure 4). At the follow-up more than one year after liver transplantation, the patient showed good signs of recovery.

## Discussion and Conclusions

HS is a genetic disease caused by congenital abnormalities in the red blood cell membrane structure. The diagnosis of HS is usually based on a positive family history, spherocytosis, jaundice, hepatosplenomegaly, and anemia. The clinical manifestations of HS are similar to those of glucose-6-phosphate dehydrogenase (G6PD) and Gilbert syndrome and are prone to misdiagnosis (5). The application of NGS is of great significance in the diagnosis of genetic diseases. It is not only beneficial for clinical diagnosis and treatment, but also important for genetic counseling. In this case, the proband, the proband's father, and the proband's younger brother presented an increase in red blood cell fragility due to unintentional mutations in the *SPTB* gene that caused anemia and jaundice. Our findings further confirm that heterozygous mutations in the *SPTB* gene cause HS.

The *SPTB* gene was first identified in red blood cells and is the main component of the membrane cytoskeleton. SPTB and red blood cell α-spectrum protein, through α^2^β^2^ allo-tetramerization, forms a dense red blood cell membrane skeleton network. This skeletal network is connected to the lipid bilayer to maintain the shape of the red blood cells (1, 16). *SPTB* gene mutations are inherited in an autosomal dominant manner. Among patients with HS in Northern Europe, *SPTB* mutations account for ~15–30% of cases. Furthermore, monoallelic *SPTB* mutations are among the most common causes of typical HS (1). Common types of *SPTB* mutations include splice sites, frameshifts, and nonsense mutations, which usually result in defects in mRNA processing and β-spectrum truncation (17). In this case, the *SPTB* gene heterozygous mutation (c.4117C>T) was unintentional, and resulted in a frameshift and a premature stop codon downstream of the C-terminal coding region of the dimer, which caused instability of the mutated mRNA and insufficient spectrin protein synthesis and affected the integrity and flexibility of the erythrocyte membrane skeleton. The main clinical manifestation of this mutation is moderate or severe HS.

In addition to red blood cells, SPTB is mainly found in the bone marrow, heart, brain, placenta, and prostate tissue and is relatively low in the liver tissue. Recent research has found that SPTB also plays an important role in liver cell regeneration and repair. The βI spectrum protein encoded by *SPTB* is reduced in hepatocellular carcinoma and cholangiocarcinoma cases, while it is expressed in normal hepatocytes and all focal nodular hyperplasia and liver adenoma (18). Homozygous *SPTB* gene mutations in newborns can cause severe transfusion-dependent hemolytic anemia, combined hyperbilirubinemia, coagulopathy, portal hypertension, massive ascites, and progressive liver failure (19). In this case, complete mitochondrial gene sequencing of the proband showed no abnormalities. Additionally, re-analysis of the data of all exons did not reveal a second single-gene disease to explain liver disease. We speculate that *SPTB* mutations may cause liver dysfunction and cirrhosis in addition to HS, and our findings expand the phenotypic spectrum of *SPTB*.

## Data Availability Statement

The datasets presented in this article are not readily available due to ethical and privacy restrictions. Requests to access the datasets should be directed to the corresponding author.

## Ethics Statement

The studies involving human participants were reviewed and approved by the Ethics Review Committee of the First Affiliated Hospital of Xi'an Jiaotong University. The patients/participants provided their written informed consent to participate in this study. Written informed consent was obtained from all participants for the publication of any potentially identifiable images or data included in this article.

## Author Contributions

Patient management, data collection, and analysis were performed by all authors listed as physicians. Further data analysis was performed by XY and XL. Histopathological examination was LC. Materials were provided by WW. The first draft of the manuscript was written by XY. All authors commented on previous versions of the manuscript, study conception and design, read, approved the final manuscript, contributed to the article, and approved the submitted version.

## Funding

This study was supported by Institutional Foundation of the first affiliated hospital of Xi'an Jiaotong University 2018QN-13, Natural Science Foundation of Shaanxi Province 2017JM8083.

## Conflict of Interest

The authors declare that the research was conducted in the absence of any commercial or financial relationships that could be construed as a potential conflict of interest.

## Publisher's Note

All claims expressed in this article are solely those of the authors and do not necessarily represent those of their affiliated organizations, or those of the publisher, the editors and the reviewers. Any product that may be evaluated in this article, or claim that may be made by its manufacturer, is not guaranteed or endorsed by the publisher.
